# Impact of COVID-19 on Sexual and Gender Minority Communities: Focus Group Discussions

**DOI:** 10.3390/ijerph20010050

**Published:** 2022-12-21

**Authors:** Jennifer R. Pharr, Emylia Terry, André Wade, Amanda Haboush-Deloye, Erika Marquez, Nevada Minority Health and Equity Coalition

**Affiliations:** 1 Department of Environmental and Occupational Health, School of Public Health, University of Nevada, Las Vegas, Las Vegas, NV 89119, USA; 2 Nevada Minority Health and Equity Coalition, School of Public Health, University of Nevada, Las Vegas, Las Vegas, NV 89119, USA; 3 Silver State Equality, North Las Vegas, NV 89031, USA; 4 Nevada Institute for Children’s Research and Policy, School of Public Health, University of Nevada, Las Vegas, Las Vegas, NV 89119, USA

**Keywords:** sexual and gender minorities, mental health, physical health, vaccine hesitancy, contact tracers, LGBTQIA+

## Abstract

Background: People who identify as sexual and gender minorities (SGM) experienced disproportionate economic and mental health issues related to COVID-19 when compared to the general population. The purpose of this study was to better understand how COVID-19 has impacted the SGM community and ways to address vaccine hesitancy. Methods: Three focus groups were conducted with 21 members of the SGM community between 5 November and 10 December 2020. A thematic analysis using the reflexive approach was applied to the transcripts of the focus groups. Results: Four themes emerged: (1) Impact of COVID-19 on the Community, (2) Perceptions of Contact Tracing and Testing, (3) Perceptions of a Potential COVID-19 Vaccine, and (4) Decreasing Vaccine Hesitancy. The most relevant subthemes were that social isolation led to anxiety, stress, and fear in the SGM community during COVID-19; resilience and adaptation were positive outcomes of the pandemic; histories of medical racism contributed to hesitancy to get tested; and specific messaging from trusted messengers may be needed to encourage SGM communities to get vaccinated. These findings support other COVID-19 research on the SGM community during the start of the pandemic. Conclusions: This study provides insight into the impact of the early stages of COVID-19 on the SGM community, highlighting the unique hurdles faced by SGM individuals with regard to contact tracing and vaccine hesitancy.

## 1. Introduction

In December of 2019, COVID-19 raised concerns around the world as patients in Wuhan, China, reported unknown respiratory illnesses [1,2]. By January 2020, as cases began to rise and spread, crossing national borders and continents, the World Health Organization (WHO) declared that coronavirus was a public health emergency of international concern [1,2]. Less than 2 months later, COVID-19 was upgraded to an international pandemic. Given its mode of transmission, experts supported wearing masks, social distancing, and proper hand hygiene to prevent COVID-19, and many states mandated stay-at-home orders to stop the spread in March of 2020 [2]. Between workplaces and schools closing, the imposition of travel restrictions, lockdown measures, significant illness, and job layoffs, life for many Americans shifted quickly and dramatically in response to the pandemic [2,3].

These changes precipitated significant economic and mental health concerns. Between March and April of 2020, 22 million unemployment claims were filed in the United States (U.S.) in an unprecedented wave of job losses [4]. Ultimately, COVID-19 led to record-high rates of unemployment, with millions of households experiencing food and housing insecurity; sexual and gender minority (SGM) individuals, communities of color, and families with children faced disproportionate impacts [4,5,6,7]. Additionally, the pandemic led to an increase in mental health disorders, such as anxiety, depression, suicide ideation, and substance use, with SGM people, communities of color, women, immigrants, and households with children indicating worse overall outcomes [8,9,10].

SGM individuals, or people who identify as lesbian, gay, bisexual, transgender, queer/questioning, intersex, or asexual, also faced severely disproportionate economic impacts related to COVID-19 when compared to the general population [7,11]. SGM individuals reported greater rates of job loss and housing and food insecurity, with SGM people of color indicating both greater infection rates and financial hardships [11,12]. These types of financial hardships are well-documented as having a negative impact on mental well-being and health [13,14,15,16,17]. In addition, minority stress and stigmatization have been linked to higher levels of mental illness among SGM individuals, and many members of the community faced disproportionate rates of mental health disorders prior to the pandemic [18,19,20,21,22,23,24,25,26,27,28,29]. COVID-19 further exasperated mental health issues among SGM people and increased depression, anxiety, stress, suicidality, PTSD, identity concealment, and COVID-19 related worry [10,30,31,32,33,34,35]. 

Before the COVID-19 pandemic, culturally competent mental health assistance was a barrier to care for many within the SGM community, resulting in greater unmet health needs than those among non-SGM individuals [36,37,38]. During the pandemic, healthcare and mental health support systems around the world faced disruption, potentially impacting SGM individuals’ health and mental health due to declines in service accessibility [37,38]. Given the pandemic-related mental health issues experienced by SGM individuals combined with the disproportionate financial and housing insecurity burdens experienced by this community during COVID-19, focus groups were conducted to further elucidate the impact of COVID-19 on the SGM community. Capturing community members’ voices and experiences was necessary to learn more about the issues, concerns, and experiences of SGM individuals during the height of the pandemic because of the significant health, employment, and economic disparities experienced by the SGM community both prior to and during the pandemic. Specifically, the purpose of this study was to understand how COVID-19 has impacted the SGM community, community members’ perceptions of contact tracing and of a potential COVID-19 vaccine, and prospective strategies to address vaccine hesitancy in the SGM community.

## 2. Materials and Methods

Because this study focused on questions of “how” or “why” rather than “how many” or “how much”, qualitative methodology was deemed appropriate. This study was part of a larger project for which the Nevada Minority Health and Equity Coalition (NMHEC) was awarded a grant to conduct education and outreach in key communities throughout the state of Nevada that had been greatly impacted by COVID-19. The larger project included seven different priority populations: Black/African American, Latinx, Asian, Native Hawaiian/Pacific Islander, Native American, SGM, and the deaf and hard of hearing community. Here we present the findings from the SGM community. Focus groups were conducted between 5 November and 10 December 2020. 

### 2.1. Focus Group Recruitment and Procedures

Flyers were sent out by community partners through social media or via email, as well as posted in some organizations’ facilities. Flyers included registration links which took participants to a Qualtrics survey where they could select a session to attend. For those who registered, emails were sent out by either staff or community partners regarding the meeting information and tips for utilizing the Zoom video conferencing platform, such as using a laptop and headphones. Reminder emails were sent out the day of the meeting, and if an email was not provided, a reminder phone call was made. For their participation in a focus group, individuals were offered a 20-dollar gift card to show appreciation for their time.

The focus groups were facilitated by at least one person from the partner organization and two staff members were present to support the facilitator. Focus groups were held via Zoom. Once the session began, participants were given a short summary explaining the purpose of the focus group and then were asked for their permission to be recorded. Participants were also reminded that their name would not be connected to any of their responses. The facilitator addressed a few ground rules, such as staying present during the meeting, limiting side conversations, understanding that all responses are valid, speaking one at a time, welcoming all perspectives, and keeping information shared by participants confidential. 

At the end of the focus groups, a link to a short demographic survey was provided in the chat box and participants were asked to complete it directly after the focus group. No identifying information was collected on this form and completion of the form was not required to receive the incentive. Focus groups typically lasted 1 h and 30 min. 

### 2.2. Focus Group Questions

Staff created a semi-structured interview of 12 questions, as well as additional prompts that guided each focus group. Questions were sent out to the community partners to solicit their input on content and phrasing. The questions served as a guide for the focus groups, but groups were encouraged to let the conversation flow naturally. These questions included topics related to how COVID-19 has impacted community members, case investigation, contact tracing, as well as opinions and planned behaviors related to the COVID-19 vaccine and vaccine hesitancy. Key questions focused on the challenges that communities have faced, specific barriers related to each community, cultural beliefs that may prevent them from getting tested for COVID-19 or taking the vaccine, and any information that could be shared with the community to address negative perceptions about testing and a potential vaccine. In addition, information was collected about important factors and messaging that should be considered when speaking with the priority populations regarding a vaccine. Participants were also asked about trusted sources for COVID-19 information in each community. Lastly, participants were asked how they were impacted by the pandemic so that the researchers could identify factors that affected the community socially, financially, at home, at work, and with regard to technology. An additional question was asked about how they were coping during that time.

### 2.3. Data Analysis

The data (audio files and notes) from the three focus groups were transcribed verbatim and subjected to a thematic analysis using the reflexive approach [39,40]. Two investigators read the transcripts independently and identified sentences and phrases meaningful to the research topic using both a deductive and inductive methods. The inductive approach was data driven, while the deductive approach was guided by the semi-structured interview guide. The coding was done manually. The initial codes were compared, and differences were resolved by discussion between the two investigators. A codebook was then created, and the investigators used the codebook to independently complete the coding of all transcripts. The final codes were then aggregated to generate broad themes for further examination and discussion. The generated themes were used to understand the perceptions of the SGM community regarding the COVID-19 pandemic. Frequency distributions for demographic data of the participants were calculated. We also conducted member-checking for validity of the results by having members of the focus group review our themes and subthemes so that they could confirm the credibility of the information and narrative account. 

### 2.4. Ethical Concerns

This study was deemed exempted by the University of Nevada, Las Vegas Institutional Review Board because no identifying information was collected. Prior to participating in the focus group, participants were provided with an informed consent statement and had to verbally consent to participate. Verbal consent was selected for anonymity to avoid linking the participants to the study through a written consent form. Participants were asked to select a pseudo name to use during the focus group discussion to conceal their identity. The demographic data collection was anonymous and could not be linked to participants or their answers. 

## 3. Results

Three focus groups with 21 participants were conducted via Zoom. Participants were from across the state of Nevada. The demographic characteristics of the participants are shown in Table 1. A majority of the participants identified as White (33%), Black or African American (29%), or Hispanic (19%); were between the ages of 18–50 (67%); had attended some college or graduated from college (71%); had a gender identify of male (71%); and rented their homes (66%). The income group reported by the largest percentage of participants was $15,000–$24,999 (29%). 

Several themes emerged from the focus groups specific to the questions around the impact of COVID-19 on the SGM community, perceptions of contact tracing and testing, perceptions of the COVID-19 vaccine, and ways to decrease vaccine hesitancy. Themes with corresponding participant quotes are provided below.

### 3.1. The Impact of COVID-19 on the Community

#### 3.1.1. Subtheme: Fear, Anxiety, Stress, and Social Isolation

Participants in the focus groups reflected most on the impact of COVID-19 on the SGM community, and there were several different concerns raised. Participants mentioned that there was fear and anxiety related to the situation, which created a lot of stress, coupled with missing out on certain experiences such as graduation or starting college. They also discussed the stress caused by not being able to visit family members who were hospitalized.

“Having your loved one in the hospital, and you cannot visit. And then when he passes, you cannot have friends over to console you, making it nearly impossible to get through your day.”

In addition, many expressed a certain level of social isolation because they could not be with a community where they felt comfortable and where they could be themselves. Some reflected on SGM people having to spend more time with unsupportive family, while several mentioned feeling like they had been put into a “box”. Over time, these issues began to have a negative impact on their overall mental well-being.

“For me, with my addiction and in my recovery, I used to isolate myself a lot. And so, in recovery, I started gaining a lot of friends… And it felt like I lost that again, and so that was almost triggering in a sense, too. So, it definitely did impact my mental health a lot more than I thought it did.”

“She is not fully out to her whole family and now she is just kind of stuck with them and she kind of has to suppress that all down because her dad… says some very nasty things that I could never repeat but you know it is directed towards the LGBT [lesbian, gay, bisexual, transgender] community… She does not have the chance to move out and be herself… Thanks to COVID, she cannot be happy or herself.”

#### 3.1.2. Subtheme: Impacts on Physical Health

Participants reflected on the pandemic’s varied impacts on their physical health. While no health impacts were discussed by some participants, others reported that they were not exercising as much as before.

“You cannot get around as much... I mean, you can choose to exercise in your house, but… those four walls tend to get smaller and smaller when you try to exercise… And it gets old after a while.”

Additionally, medical visits shifting from in-office to virtual visits was a topic of focus, and this seemed to be a positive change for some participants.

“I actually prefer all the telehealth. For me, I work Monday to Friday, so business hours for most offices. And it is hard to go before work or after work or even on lunch because it will make me late to anything, so doing it with Zoom or over the phone, I can just go into my car real quick, get it done, [and] go back to work without have to worry about missing hours or having to call in or anything like that.”

#### 3.1.3. Subtheme: Disability and Co-Morbidities as Additional Challenges

Some participants expressed that members of the SGM community were likely to be at an elevated risk for contracting COVID-19 compared to other groups due to high rates of underlying conditions, such as being immunocompromised. Other participants reflected on how having disabilities or other co-morbidities made living during the pandemic especially challenging. During the “stay-at-home” period when one of the few options to leave one’s home was to go outside, some were not able to easily do so due to their disabilities, leading to a sense of isolation.

“I’m disabled, and it has been really rough to get outside, I cannot go.”

Additionally, it became more challenging to find care for other co-morbidities during the pandemic.

“I am currently battling cancer, and so I am immunocompromised. So being in a pandemic, and battling cancer, and being immunocompromised, is quite an ordeal.”

#### 3.1.4. Subtheme: Economic Challenges

Among participants who were not facing pandemic-related lay-offs or job loss, some mentioned that their employers would fire people if they called in sick, forcing employees to potentially go to work infected because they were in need of money. Many participants also commented that their families or themselves experienced the loss of work, which also made COVID-19 very challenging. This was especially true for those in the performance industry.

“I’m a dancer so a lot of those opportunities have been shut down, especially for the performers on the [Las Vegas] Strip. I would say [the impact of job loss] has been more so in the creative arts realm.”

#### 3.1.5. Subtheme: Resilience and Adaptation

Although much of the discussion around COVID-19 focused on challenges, participants did discuss how they were adapting to the situation and finding resilience. Participants talked about trying new things, such as hobbies, with a sense of hope and optimism.

“I went and got myself a sewing machine and… I am sewing everything now. I am fixing my own clothes, I am making masks… and that is all because of COVID.”

“Instead of doing phone interviews [for my podcast], I do them all via Zoom and record them so it is… on the Facebook page, but then it is also audio. So, it makes it more engaging, and, you know, it gains a… broader audience… [by] bringing it visually instead of just hearing.”

“I am having my first doctor’s appointment on Zoom… so I am excited to try it out.”

### 3.2. Perceptions of Contact Tracing and Testing

When discussing different methods of tracking COVID-19, the majority of participants did not have any concerns. They expressed that they were familiar with methods that are used for sexually transmitted infections (STI’s), including HIV. However, some participants still expressed apprehension about using an online application that obtained personal information and could potentially track one’s location. There was also confusion about how COVID-19 tracking would be accurate, as it relies on people self-reporting. Three major themes emerged: cultural attitudes of Black/African American and Latinx/Hispanic SGM people toward getting tested for COVID-19, histories of medical racism, and the stigma associated with having a positive test.

#### 3.2.1. Subtheme: Cultural Attitudes Contributing to Hesitancy to Get Tested

Participants talked about how culture influences decisions about seeking medical care and how that might guide Black/African American and Latinx/Hispanic individuals’ decisions about getting tested for COVID-19.

“It is harder for the Black and Hispanic communities to get on board with testing just because there are so many barriers in healthcare and access to healthcare. and when you ask people in those communities about where they receive healthcare, a lot of them are very disjointed. It is kind of random. They only go to the doctor when they are sick…it is going to be really important for the medical community and organizations that serve those communities to reach into those communities and create messages that resonate—not just that it is safe to get vaccinated and they can give them the science behind it, like someone said, but also that it is a safe place to come and get vaccinated where you will not be judged [and] it will not cost you”.

“I am a Mexican–American, OK? And if you are not [from a] Latino family, you know some folks be like… all you got is just a cough, a sneeze, mild indigestion. Just put on some Vicks, and you are good. [LAUGHS] They would be saying stuff like that. And thinking that, oh, it is not COVID, it is just you being you, or your allergies, or something else.”

#### 3.2.2. Subtheme: Histories of Medical Racism Contributing to Hesitancy to Get Tested

Participants also reflected on how past medical racism contributes to hesitancy among minority SGM people, and particularly Black/African American community members, toward getting tested for COVID-19 or engaging in contact tracing.

“So, you know, historically mentioning the Tuskegee Airmen… Black bodies have been used in medical experiments, Black women, Black men, and… is there anything related to the LGBTQ [lesbian, gay, bisexual, transgender, queer] community that would prevent folks from wanting to get tested or do contact tracing that is kind of related to the same thing?”

“I think it carries over, it is kind of passed down. I know statistically speaking African Americans are the most timid to go to the doctors, they are afraid that they cannot trust the information because of such things that have happened historically. So, I believe it is carried into the LGBTQ community for Black bodies because you cannot separate your Black from your LGBTQ so they kind of go hand in hand, so I think that those cultural beliefs, that have been passed down historically—they are continuing.”

#### 3.2.3. Subtheme: Stigma against Positive Tests and Testing

Stigma against testing positive for COVID-19 was also identified as a barrier to getting tested. Concerns centered around quarantining the household if a person tested positive and the fear of being ostracized from the community if others found out that a person had tested positive for COVID-19. One participant talked about the backlash they received after announcing on social media that they had been tested for COVID-19.

“I’m scared to get tested. Because if I’m tested, and I get positive, then like everybody in my household is shut down.”

“COVID is also becoming a taboo. And even in the gay community, where, like, they would not want to tell you because they’re ostracized from the community, even if it is 2 weeks or a month.”

“When they first announced that they were doing free COVID testing, you know. I made it publicly known on Facebook that I went and got tested and I received so much backlash. Saying, why did you go get tested?”

### 3.3. Perceptions of a Potential COVID-19 Vaccine

While some participants were in favor of being vaccinated, some participants in the focus groups expressed concerns about taking the COVID-19 vaccine, especially during its initial rollout, as they did not want to be the first to try it. The political climate was also identified as a barrier to the COVID-19 vaccine.

#### Subtheme: Political Climate Influences on COVID-19 Vaccine and Informational Hesitancy

The political climate was identified as an influence on COVID-19 precautions and vaccines. However, participants felt that things might get better since a new administration had been elected.

“I just wanted to ask [is there] specifically anything from a cultural standpoint from the LGBTQ community that might prevent us from getting the vaccine? Yeah, while Trump is still in the office… I would not think about it [vaccine] now.”

“Everything that I was worried about was kind of what the president was saying, or the ex-president, because he was kind of just like not wanting diverse people to get help, but now that we have a bit of a brighter future. I believe that there should not be as many problems.”

### 3.4. Decreasing Vaccine Hesitancy

Participants were asked what they would recommend to reduce vaccine hesitancy within the SGM community. Four themes emerged as important to reducing hesitancy, including clarifying what type of information to present to SGM individuals and what questions should be answered, increasing community understanding about trusted sources of vaccine information, addressing resources needed to make the vaccine accessible, and creating potential messages to encourage the SGM community to get vaccinated. 

#### 3.4.1. Subtheme: Information Needed to Trust the Vaccine

Participants wanted honest information to be provided to the community, presenting both the pros and cons of the vaccine. They also highlighted questions that they thought needed to be answered for SGM individuals to participate in COVID-19 vaccination programs.

- How was the vaccine developed so quickly?
- If I am taking hormone treatments, can I still get the vaccine?
- If I have HIV is the vaccine less likely to work?

#### 3.4.2. Subtheme: Trusted Sources of Vaccine-Related Information

Participants discussed the sources that they trusted for information about the COVID-19 vaccine. These included:

- Centers for Disease Control and Prevention
- Family/friends who are health professionals
- World Health Organization
- News on TV or Radio
- Social media influencers

#### 3.4.3. Subtheme: Making the Vaccine Accessible

Participants were asked what would be necessary in order for people to have access to the vaccine. The following were the top recommendations to ensure that people in the community could obtain the vaccine.

- Needs to be free
- Need to be offered in a close, convenient location, such as:
  ○ Clubs
  ○ Common gathering spaces like recreation centers
  ○ Places that specifically serve the community
  ○ The LGBTQ Center

#### 3.4.4. Subtheme: Potential Messages for the Vaccine 

Participants were also asked what type of messages would be most likely to encourage SGM community members to receive the vaccine. The main messaging recommendations were:

- Do not allow anybody to intimidate or force them to do anything; this is a choice
- See friends and family again by getting vaccinated
- Vaccine messaging should be relevant to the SGM culture without stereotyping

“I was thinking it is kind of like the, you know, the PrEP messaging. So, the vaccine … could mean that people could go to clubs and be in crowds and hook up and not worrying about catching COVID so, I would say it gets you back to normal. It gets you back to normal.”

“[The messages] just seem very redundant and stale, so I would just like to see something more creative, expressive, colorful, not stereotypical, do not get me wrong, but something that just encapsulates us, but all different versions of us. Not the same version of us, either.”

## 4. Discussion

The results of this study show that the SGM community has been uniquely impacted by COVID-19. In Nevada, a relatively high proportion of the population (5.5%) self-represents as a sexual or gender minority individual, and 51% of SGM Nevadans are from diverse racial or ethnic backgrounds [41]. This intersectionality of identities was present in the themes identified by focus group participants. For example, job loss and medical racism were two of several issues cited by participants.

Notably, SGM people of color were two times as likely as white non-SGM individuals to receive a positive COVID-19 test result during the early stage of the pandemic [11]. This is significant, as SGM people of color faced disproportionate rates of pandemic-related lay-offs or furloughs, leading to financial hardship in acquiring basic needs, and 26% expressed concern about paying for housing [11]. Due to factors including lack of workplace protections, discrimination, and under-funded schools, SGM people of color experience disproportionate rates of poverty and unemployment and are some of the most vulnerable employees in the workforce [42]. These concerns were highlighted within the focus groups.

Racism and violence have long been present in medicine and healthcare in the U.S., resulting in many inequities such as unequal access to care and the barring of Black Americans from pursuing medical degrees [43]. While most focus group participants were used to contact tracing, as it has long been used within the SGM community for sexually transmitted infections such as HIV, others were wary about being part of a biological database. Some described racism and homophobia in the medical field as a constraint to getting tested for COVID or participating in contact tracing, naming both the Tuskegee untreated syphilis study and ongoing structural barriers to care and discrimination in healthcare as injustices preventing communities of color from accessing healthcare [44,45]. 

Participants also cited racism within the highest levels of government as impacting health outcomes during the pandemic, describing the Trump administration’s handling of COVID-19 as harmful to communities of color and a barrier to vaccination uptake. In 2018, a survey by the Associated Press-NORC Center for Public Affairs Research revealed that 57% of adults—including 80% of Black Americans, 75% of Hispanic/Latinx Americans, and almost half of white Americans—believed that President Donald Trump was a racist, with many feeling that he had exacerbated racism against communities of color [46,47]. This sentiment was echoed within focus groups and was named as a barrier to receiving the vaccine while he was in office.

Additionally, focus group participants reported that they, or their loved ones, had experienced job loss or the threat of it, with entertainers particularly impacted by pandemic job-related disruptions. Nevada was one of the states hit hardest by the pandemic, as there is a higher percentage of Nevadans employed in the hospitality, leisure, and retail spaces [48]. Despite casinos reopening, many positions have not returned, leaving experts to warn that Nevada’s economy may take years to fully recover [49]. Respondents said that business closures affected not only workers, but also those with disabilities/comorbidities and those who relied on community supports to combat stigma-related alienation and isolation. 

COVID-19 disproportionately impacted people with disabilities in many ways, including accessing healthcare, education, employment, and mental health services [50]. For example, mask mandates severely hindered communication for those with hearing impairments, as lipreading became impossible. Further, business closures and stay-at-home mandates deprived many SGM individuals of their social supports, such as those found in resource centers and clubs, leaving many stuck with intolerant family or roommates [51]. Focus group participants cited these apprehensions, as well as stigma- and isolation-related negative impacts on mental well-being over time. This is particularly concerning given this population’s unduly high rates of discrimination-related mental health issues such as anxiety, stress, depression, and suicidality [52,53,54,55,56,57,58].

While there are significant barriers to COVID testing, contact tracing participation, and vaccine uptake, this study also revealed several silver linings. Firstly, participants illuminated strategies to address vaccine and testing hesitancy, such as the need for public health and health communication practitioners to recognize that SGM individuals comprise a highly diverse community. Accordingly, pandemic-related materials targeting the SGM community should be representative and applicable, with one participant recommending that advertisements showcase how vaccination uptake can foster a quicker return to socialization. Another participant highlighted campaigns surrounding pre-exposure prophylaxis (PrEP) as effectively reaching and impacting its priority audience. This observation is backed by data: Chicago’s PrEP4Love campaign was sex-positive and equity-focused, featuring individuals of all genders and backgrounds. Thanks to its cultural competency and innovative messaging to target vulnerable populations, the campaign successfully reached millions of people [59].

Lastly, participants expressed community members’ resilience and adaptability. Whether learning a new hobby or embracing new technologies, participants found ways to find some brightness in unprecedented times. While this is a significant finding, it should in no way obscure or minimize that resilience is a concept that must be situated within the context of structural and social determinants of health [60,61]. Given this community’s diversity, with many members facing intersectional oppressions, individual determinants are insufficient. 

### Limitations

As with any study, this study does have limitations. Participation was not random; therefore, there could be something different about those who participated in the focus groups compared to those who chose not to participate. Additionally, participants were only from Nevada, and perceptions of SGM people from other states may have been different due to differences in stay-at-home requirements, unemployment rates, state laws protecting SGM people from discrimination, or the acceptance of SGM community members. This could limit the generalizability of the results. In addition, given that the majority of the focus groups were held online, it excluded those who do not have access to technology. 

## 5. Conclusions

This study provides insight into the impacts at the height of COVID-19 on the SGM community, highlighting the unique hurdles faced by SGM individuals with regard to contact tracing and vaccine hesitancy. While SGM community members were adversely impacted by social isolation, which contributed to stress and anxiety, they also found resilience and the ability to adapt during the early months of the pandemic [62]. Finding avenues for social inclusion and social connection during stay-at-home mandates were important for SGM peoples’ health and well-being, especially for those who had to return home to live with unsupportive family members [63,64,65]. Contact tracers were not a new experience for our (male) participants, as they were familiar with contact tracing for other diseases such as those that are sexually transmitted. Participants provided insight into important messaging for the SGM community to help overcome vaccine hesitancy. This study also suggests that public health practitioners must be culturally competent and committed to addressing historical and ongoing inequities to improve pandemic-related health outcomes within the SGM community. Implications of this research for policymakers include understanding the on-the-ground impact of policies on vulnerable populations who might be more at risk for mental health issues, unemployment, social isolation, and discrimination. Additionally, policymakers need to understand the unique needs of diverse constituents in order to effectively communicate with them during times of emergency, such as a global pandemic.

## Figures and Tables

**Table 1:** Demographic characteristics of the participants.

Demographics	Number = 21
Race/ethnicity	
Black/African American	28.5%
Hispanic	19.0%
Native Hawaiian	5.0%
White	33.3%
Prefer not to Answer	14.2%
Age	
18–30	28.6%
31–50	38.1%
51–64	23.8%
65 or older	4.8%
Prefer not to Answer	4.8%
Education Level	
Less than 9th grade	0.0%
9th to 12th grade, no diploma	0.0%
High School Diploma or GED	9.5%
Some college, no degree	42.9%
Associate’s Degree	28.6%
Bachelor’s Degree	19.0%
Graduate/Professional Degree	0.0%
Prefer not to Answer	0.0%
Gender	
Male	71.4%
Female	23.8%
Transgender (MTF)	4.8%
Transgender (FTM)	0.0%
Gender fluid/Non-binary	0.0%
Prefer not to Answer	0.0%
Income	
0	4.8%
<14,999	0.0%
15,000–24,999	28.6%
25,000–39,999	9.5%
40,000–54,999	14.3%
55,000–79,999	4.8%
80,000–109,999	9.5%
110,000+	9.5%
Prefer not to Answer	19.0%
Home	
Rent	66.7%
Own	23.8%
Prefer not to Answer	9.5%

## Data Availability

Not applicable.
